# The Need for Inducing Tolerance in Vascularized Composite Allotransplantation

**DOI:** 10.1155/2012/438078

**Published:** 2012-10-31

**Authors:** Kadiyala V. Ravindra, Hong Xu, Larry D. Bozulic, David D. Song, Suzanne T. Ildstad

**Affiliations:** ^1^Department of Surgery, Duke University Medical Center (DUMC) 3512, Durham, NC 27710, USA; ^2^Institute for Cellular Therapeutics and Jewish Hospital, University of Louisville, 570 South Preston Street, Suite 404, Louisville, KY 40202-1760, USA

## Abstract

Successful hand and face transplantation in the last decade has firmly established the field of vascularized composite allotransplantation (VCA). The experience in VCA has thus far been very similar to solid organ transplantation in terms of the morbidity associated with long-term immunosuppression. The unique immunological features of VCA such as split tolerance and resistance to chronic rejection are being investigated. Simultaneously there has been laboratory work studying tolerogenic protocols in animal VCA models. In order to optimize VCA outcomes, translational studies are needed to develop less toxic immunosuppression and possibly achieve donor-specific tolerance. This article reviews the immunology, animal models, mixed chimerism & tolerance induction in VCA and the direction of future research to enable better understanding and wider application of VCA.

## 1. Introduction

Two areas of transplantation that posed significant obstacles to clinical application were vascularized composite allotransplantation (VCA) and donor-specific tolerance. However, over the last decade, it is heartening to note the progress that has been made in both of these fields. VCA has achieved acceptance in the field of transplantation [1] and promises to grow exponentially in the next few years. In the last 5 years there have been prospective investigational studies of donor bone-marrow infusion in living donor renal transplant recipients which have successfully induced donor-specific tolerance [2–5]. This new development has the potential for a wider application.

## 2. Immunology of VCA

Clinical feasibility of VCA has been established with the long-term success of hand and face transplantation. Over 50 hand and 14 face transplants have been performed worldwide with excellent outcomes [6]. The successful transplantation of these skin-bearing structures has been possible with the availability of potent immunosuppression. The vast majority of these recipients were managed with lymphocyte-depleting induction therapy [7] and triple drug maintenance immunosuppression (tacrolimus, MMF, and prednisone). T-cell depletion through antibody-mediated induction therapy is routinely used to promote long-term graft survival in solid organ transplantation. The most commonly used agents include antithymocyte globulin (ATG) and Campath-1H [8]. The majority of patients undergoing VCA have received T-cell depleting induction therapy [7]. Despite this aggressive immunosuppressive therapy, episodes of acute rejection have been recorded in 85% of hand and 54.5% of face transplant recipients in the first year after the transplant [9–11]. Thus the incidence of acute rejection following VCA transplantation is significantly higher than that seen currently with solid organ transplantation—the overall incidence of acute rejection within the first year after renal transplantation is now less than 15% [12]. 

### 2.1. Immunology of VCA: VCA Is Not One Single Tissue. 

VCA is composed of skin, muscle, vessels, nerves, tendon, bone, and so forth—each with differing immunogenic potential. Skin is probably the most immunogenic of all human tissues [13]. Lee et al. demonstrated that a whole limb allograft elicits a less intense alloimmune response as compared to each of its individual components [14]. This notion has been significant in the success of a whole limb allotransplantation compared to an isolated skin allotransplantation [15]. Several theories have been put forward to explain this and include (1) the vascularization of the skin arises from the donor in the whole limb versus the recipient in the isolated skin graft; (2) the occurrence of a consumption phenomenon when the host immune system is exposed to an excessive antigen load. A definitive immunological reason is yet to be elucidated [16].

In addition, the other theoretical advantage of VCA is the potential to transplant vascularized bone marrow present in the skeletal component of the allograft. The bone marrow is transplanted with its microenvironment. This has been postulated to confer an immunomodulatory effect that could lead to an improved long-term graft survival [17]. Although this concept has been established in experimental studies, there is paucity of data to support this in the clinical setting [18, 19]. Not surprisingly, graft-versus-host disease (GVHD)—a common occurrence with bone-marrow transplantation—has not been reported following VCA [7]. Notably, while VCA in the rat contains hematopoietic tissue, most bones in human VCA are not hematopoietic.

### 2.2. Acute Rejection in VCA

The high antigenicity of skin can be traced to the high proportion of potent antigen-presenting Langerhans cells. These and skin keratinocytes express MHC class I constitutively and upon stimulation present MHC class II, intercellular adhesion molecule 1 (ICAM-1), and proinflammatory cytokines. In addition, skin bears similarity with solid organs such as lung and intestine which have the highest rates of acute rejection [20, 21]. Skin biopsies from transplanted limbs have shown infiltration by CD3 positive T cells: both CD4 and CD8 subtypes and a minority of CD4 and CD8 negative cells [22]. During rejection, there is an increased expression of CD68, FoxP3, and indoleamine 2, 3 dioxygenase. Adhesion molecule expression is upregulated upon rejection—ICAM-1 and E-selectin correlated with severity of the rejection process [22]. 

Clinically, episodes of rejection are manifested by the appearance of characteristic cutaneous lesions—rash, edema, vesiculation, desquamation, necrosis, and ulceration [23]. Atypical rejection with reddening of palm and nail changes has occasionally been seen [24]. Biopsy of the skin (often protocol based without visual changes) remains the gold standard for detection of acute rejection. Acute rejection manifests initially as mild perivascular lymphocytic/mixed cellular infiltrate in the dermis. With an increase in the severity of rejection, there is an involvement of skin adnexal structures and epidermis that may lead to frank necrosis if left untreated. The Banff 2007 working classification is the currently used system to classify rejection in VCA [25], including Grade 0: no or rare inflammatory infiltrates; Grade I: Mild perivascular infiltration and no involvement of the overlying epidermis; Grade II: moderate-to-severe perivascular inflammation with or without mild epidermal and/or adnexal involvement (limited to spongiosis and exocytosis) and no epidermal dyskeratosis or apoptosis; Grade III: Dense inflammation and epidermal involvement with epithelial apoptosis, dyskeratosis, and/or keratinolysis; Grade IV: frank necrosis of epidermis or other skin structures.

There are two aspects that are well established in solid organ transplantation that are yet to be clearly delineated in VCA. These are the roles of HLA antibodies and the occurrence of chronic rejection. In renal transplantation, humoral rejection is diagnosed by the presence of (1) histological injury—neutrophils in capillaries, acute tubular injury and fibrinoid necrosis; (2) evidence of antibody interaction with tissue—C4d deposition in peritubular capillaries; (3) serological evidence of antibodies to donor HLA (DSA). The occurrence of this triad is clearly related to organ dysfunction. The incidence and occurrence of humoral rejection in VCA have not been studied. Although C4d deposition has been documented in VCA literature, it has been described in the absence of donor-specific antibodies and histological tissue injury [26].

Similarly, chronic rejection is poorly defined in VCA. Histological and clinical features indicative of chronic injury in VCA include vascular narrowing, loss of adnexa, skin and mucosal atrophy, fibrosis of deep tissue, myointimal proliferation, and nail changes [11]. Transplant vasculopathy and features suggestive of chronic rejection have been induced after multiple untreated episodes of acute cellular rejection in a rat hind-limb allotransplantation model [27]. Graft vasculopathy has been described in hand transplant recipients and has been associated with graft loss in one patient [28]. Novel methods such as the use of ultrasound biomicroscopy to evaluate vessel wall thickness in VCA grafts have been proposed to enable early detection of graft vasculopathy [28]. However, the etiopathogenesis, incidence, risk factors, and management of this entity in VCA remain to be defined.

### 2.3. Clinical Results in VCA

Functional outcomes after hand transplantation have been excellent. In the report of the international registry on hand and composite tissue transplantation [6], protective sensibility was restored in 100% patients, tactile sensibility in 90%, and discriminative ability in 84%. Most patients are able to perform daily activities one year after transplantation and their quality of life was significantly improved. The majority of them returned to work eventually. 

In contrast, functional outcome assessment in face transplant recipients is more difficult to standardize due to the uniqueness and complexity of the defect in individual patients. Results from the early recipients are very encouraging: the first four patients were able to eat, drink, and speak within 10 days of transplantation [29]. As new functional units such as tongue and lacrimal gland are added to the facial allograft, these outcomes are likely to improve even more.

### 2.4. Burden of Immunosuppression

Unlike solid organ transplantation, greater scrutiny has been placed on immunosuppression-induced complications in VCA recipients. This is largely appropriate as VCA has been deemed life enhancing as opposed to a life saving intervention. On this basis, recipient selection has been very stringent thus far in VCA. The majority is physically healthy individuals suffering from severe tissue defects of the face or limbs [30]. Despite the thorough vetting of potential recipients, the observed postoperative complications have largely mirrored those described in solid organ transplantation.

Metabolic complications have been reported in 69% of hand recipients and include diabetes, hypertension, and renal dysfunction including the need for renal replacement therapy in 1, Cushing's syndrome and aseptic vascular necrosis of both hips needing replacement [6]. The majority of recipients developed infectious complications: CMV infection occurred in 10/33 hand recipients. Interestingly, severe CMV infection was noted in two of the first four face allotransplant recipients [31]. Posttransplant lymphoma and basal carcinoma of nose have been reported in hand recipients [6].

Graft loss has been reported in the hand transplant literature: 7 patients from China due to an inadequate immunosuppression; 3 patients from the West from the cessation of immunosuppression, transplant vasculopathy, and bacterial infection [6]. More concerning is the reported mortality following face allotransplantation: 2 of 17 recipients to date have died: one Chinese recipient died 2 years after the procedure from an unknown cause and the world's first recipient of simultaneous face and hand transplant died at 2 months [32].

Clearly, there is a significant price to pay for the immunosuppression currently essential for the successful VCA. While we strive to gain better understanding of the immunology of VCA, the results thus far urge us to find ways to minimize the need for immunosuppression in these recipients. It is time to consider a clinical application of tolerance data accrued from animal experiments and the clinic.

## 3. Clinical Success in Organ Transplantation Tolerance

Induction of chimerism has been shown to be a reproducible method to induce tolerance in the laboratory. Studies published in 2008 supported strategies to achieve tolerance by donor bone-marrow infusion in living donor renal transplant recipients. The limitations of the studies were success only in HLA matched pairs in one study [2] and only short lived chimerism (undetectable after 2 weeks) and engraftment syndrome in the other [3]. A recent approach using a different strategy has reported durable chimerism (5 of 8 patients at 15−30 months) and tolerance induction in mismatched living donor renal transplant recipients [4]. The technique is based on nonmyeloablative conditioning using cyclophosphamide, fludarabine, and 200 cGy of total body irradiation (TBI). The renal transplant is followed by infusion of a bioengineered mobilized cellular product enriched for hematopoietic stem cells and facilitating cells. The facilitating cells have been previously demonstrated to promote engraftment without an increase in GVHD [33]. Based on the experience in the first 4 patients, the appropriate dose of *αβ* T-cell dose has been defined. Subsequent patients have demonstrated durable multilineage chimerism and have been successfully weaned off all immunosuppression. None of the recipients developed GVHD or donor-specific antibodies. Some of the complications that have been reported in the above tolerance induction studies include engraftment syndrome with reversible acute kidney injury [34] and the loss of renal grafts from rejection and viral sepsis induced vascular thrombosis [4, 34]. 

Thus this approach appears very promising as a potential way forward in solid organ transplantation. But, more importantly, it may hold important lesions to enable a wider application of VCA. The reluctance on the part of the plastic and reconstructive surgeons in embracing VCA is the fear of long-term complications that are part and parcel of conventional immunosuppression. Future refinement of the above-mentioned tolerance strategies to enable use with deceased donor transplantation, further elucidation of the mechanistic components of these studies and a longer-term followup of the “tolerant patients” could help persuade reconstructive surgeons to use VCA. 

## 4. Animal Models in VCA Study

It is known that the current challenge in the widespread clinical applicability of VCA is the toxicity of high-dose postoperative immunosuppression. The use of preclinical animal models is essential in developing novel cellular immune-reduction therapies that will ultimately make VCA a safer and viable treatment option. Both small and large animal models have been employed in studying VCA immunology thus far. 

### 4.1. Small Animal Models

Small rodent animals are widely preferred for VCA studies for certain reasons. Logistically, such animals are relatively inexpensive and easy to maintain and handle. Biologically, these animals have short lifespans and accelerated reproduction rates that allow for VCA-related immune activities to be observed along varied time points of the life history [35]. VCA is complicated due to the disparate antigenicities of the composite tissues. Small rodent models can be an invaluable tool to investigate these various tissue antigenicities, and rodent surgical models serve well to evaluate acute and chronic rejection events postoperatively [36]. Additionally small rodents are widely used as functional models to study nerve regeneration, a unique challenge to VCA [37].

Mice and rats are the predominant small animal models used in VCA studies. The mouse model is a more valuable tool for basic immunologic research due to the numerous genetic variations of inbred and knockout strains and the commercially available genetic probes and antibodies [35, 36]. Mouse models are commonly used to study specific tolerance induction therapies. In contrast, the rat model has been more prevalent in functional studies. The rat hind-limb allograft model is a hallmark in evaluating the post-operative function of composite allografts and rejection events.

However, there are limitations of small animal models as it pertains to VCA. The most significant obstacles have been the technical challenges during microsurgery. Dissection and microanastamosis in small vessels in mouse models are problematic due to the fragility of the vessel walls [38, 39]. Similar microsurgical difficulties limit rat VCA-related models. Yet novel microsurgical techniques are in development to improve and expand VCA research potentials using mouse and rat models.

Despite the technical limitations of small animal models, such animals continue to be a cornerstone of VCA research. Various composite allografts have been performed in mice and rats in the past decade, including hind-limb transplants, bone-marrow transplant (BMT), a hemiface allograft, and even an allograft of the groin region has been performed [40, 41].

### 4.2. Large Animal Models

Immune-reduction protocols that have been successfully demonstrated in small rodent models are further evaluated in large VCA animal models for efficacy and safety before clinical trials in humans. Large animal models allow for immune-reduction therapies to be further examined in more complex biological systems that are more realistic and representative of the human immune system [35, 42–45]. Models that have been used related to VCA thus far include canines, swine, and nonhuman primates. In particular swine and nonhuman primates have a clinical relevance presenting MHC antigens similar to humans. Nonhuman primates are highly preferred in pre-clinical human immunologic studies due to the genetic similarity between primates and humans. Certain species of macaque primates are also favored for they are relatively small in size and demonstrate acceptable homology to cross react with most human immune molecules [35].

Tolerance of VCA can be induced in small rodent models, but tolerance protocols established in rodents are difficult to translate to preclinical large animal models and eventually clinical human trials. In contrast to the isolated, pathogen-free lab rodents, large animal models are exposed to uncontrolled environmental factors over longer lifespans, resulting in immune-reduction protocols that are challenging to stabilize. Further, unlike rodent models, the complex immune system in large animals requires significant potent doses of immunosuppression for graft survival in unrelated donor/recipient pairs [46]. Examples of successful immune-reduction protocols in rodent models that are more difficult to demonstrate in larger animals include limb allografts and face transplant models. Both limb and face transplants are more difficult to replicate in large animals due to the higher doses of post-operative immunosuppressants involved and the differing responses of large animals to immune-reduction protocols and methods established in mice [47, 48]. Consequently direct translation of animal protocols to human clinical trials still remains daunting due to the toxicities that may be induced by concentrated post-op immunosuppressant requirements [35, 42, 43]. Thus limitations in large VCA animal models are not purely technical. The use of animal models, both small and large, in VCA research has yielded significant progress and will continue to do so. Tolerance induced via mixed chimerism has demonstrated promise in minimizing, even eliminating, postoperative immunosuppressants, enabling VCA as a widespread clinical option.

## 5. Mixed Hematopoietic Chimerism and VCA Tolerance

A major factor limiting VCA is the requirement for lifelong immunosuppression and the toxicities associated with the use of these agents [9]. Virtually all expected complications associated with the use of chronic immunosuppression, including renal failure and death from infections, have occurred now in recipients of VCA [11]. Efforts to immunomodulate the VCA graft and recipient to induce donor-specific tolerance would be transformational in organ and VCA transplantation. Immunological tolerance would achieve permanent VCA survival and abrogate the need for chronic immunosuppression. 

### 5.1. Conditioning for Induction of Mixed Chimerism

The establishment of donor hematopoietic chimerism in organ transplant recipients leads to donor-specific tolerance [49–52]. Chimerism refers to a state of a conditioned recipient in that the donor hematopoietic stem cell engrafts and produces multiple lineages of blood cells. A new immune system including that of the donor, therefore, is established in the recipient. The tolerance associated with chimerism is permanent, stable, and not easily broken [53, 54]. Immunosuppression is not required to prevent graft rejection once chimerism is present. Chimerism is the only approach that has been generalizable to all species tested, including humans [2–4]. There are two types of chimeras: full chimerism, where the donor hematopoietic system totally replaces the recipient system, and mixed chimerism, where the donor and recipient HSC coexist. To establish a full donor chimerism, the recipient's entire hematopoietic system is ablated by lethal conditioning and replaced by the donor system. In 1985, Ildstad et al. [53] reported that mixed allogeneic chimerism induces tolerance to donor-specific skin grafts. Mixed chimeras exhibit superior immunocompetence due to the presence of recipient antigen-presenting cells to which lymphocytes of both recipient and donor origin are restricted [55]. However, in this pioneer study, mixed chimerism was established with ablative irradiation and transplantation of a mixture of T cell-depleted host and donor BMC. The application of mixed chimerism to induce tolerance in transplantation has been limited by the side effects associated with myelotoxic conditioning. As a result, we and others developed clinically relevant reduced-intensity conditioning to establish chimerism in animal models [56–61]. This critical paradigm shift allowed for the development of reduced-intensity immune-based conditioning approaches to establish mixed chimerism which has been successfully translated to the clinic [62]. The immunomodulation of the host-versus-graft immune response could provide a novel form of conditioning to establish chimerism and may completely eliminate the need for TBI and myelosuppressive conditioning agents. Recently, a mixed chimerism was shown to be established by a nonmyeloablative conditioning with TBI as low as 300 cGy in an allogeneic rat model [51] and 100 cGy in a mouse model [63].

### 5.2. The Association between Mixed Chimerism and VCA Tolerance

Mixed chimerism induces donor-specific tolerance to virtually all the organs or tissues tested including skin, heart and lung, kidney, intestine, pancreas, islets, and composite tissue allografts [51, 64–66]. In an earlier study, 950 cGy ablative TBI was used in a rat model as conditioning for chimerism followed by donor hind-limb transplantation [65]. Their results showed stable chimerism and reliable limb allograft survival. However, a safe and reliable method to facilitate the induction of mixed hematopoietic chimerism for VCA tolerance is needed. Rahhal et al. [51] had recently reported that the long-term acceptance of VCA could be induced by mixed chimerism established by nonmyeloablative conditioning with TBI as low as 300 cGy combined with a short course of immunosuppressive therapy (anti-*αβ*-TCR mAb, FK-506, and antilymphocyte serum). The BMT conditioning strategies based on costimulatory blockade of CD28 or CD40 ligand in combination T-cell depletion and low doses of irradiation have also reported to induce long-term acceptance of VCA in rat [64] and to prolong VCA survival in mouse [67]. The advantage of nonmyeloablative conditioning is that the recipients will survive from their autologous reconstitution of self-stem cells if the BMT fails to take. The optimal level of donor chimerism in tolerance induction for VCA was investigated using an MHC incompatible rat model and a reduced-intensity conditioning [68]. The chimerism level correlated positively with the incidence of GVHD and long-term CTA. Levels of 20–50% donor chimerism at day 28 were optimal for VCA acceptance with minimal or no GVHD in this rat model. Higher levels of donor chimerism were also found to be associated with VCA acceptance with nonmyeloablative conditioning of anti-*αβ*-TCR mAb, FK-506, and anti-lymphocyte serum and 300 cGy TBI for BMT [51]. There was a correlation between higher levels of donor chimerism at one month after BMT and graft acceptance in animals from all groups that accepted flap allografts (38.6 ± 2.1%) compared to animals that rejected their flaps (18.9 ± 3.6%). 

### 5.3. The Vascularized Bone-Marrow Transplant in VCA

One unique feature that distinguishes VCA from other transplants is the presence of its own hematopoietic microenvironment and supportive stromal cells from accompanying donor bone. Bone-marrow-derived cells, especially plasmacytoid precursor dendritic cells (p-preDC) and the regulatory T cells (T_reg_) they generate, maintain self-tolerance through regulatory feedback loops. They also show promise as a cell-based therapy to promote allograft acceptance. Bone marrow has long been appreciated to possess immunomodulatory properties [50, 69–72]. Therefore, the vascularized BM transplant (VBMT) model has been developed as a better source for hematopoietic cell reconstitution than transplantation of cellular BMC [73]. This model promotes long-term mixed chimerism and tolerance [74] with a decreased incidence of GVHD [75]. The sources of the vascularized bones tested have been hind limb [76, 77], sternum [78], femur [79], maxilla [80], and ilium [81]. 

### 5.4. The Timing between Chimerism Induction and VCA

**Figure 1:**
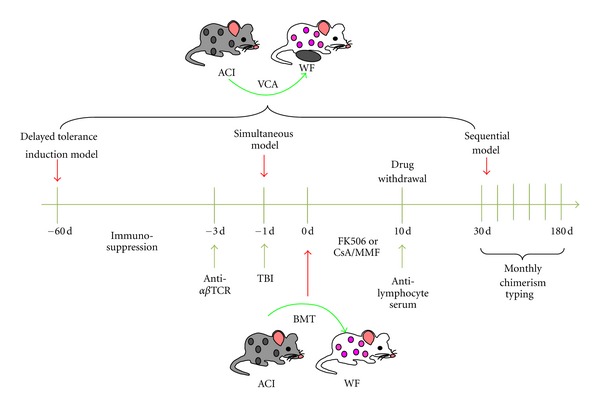
Schema for inducing VCA tolerance.

The timing between BMT and VCA is also an important and clinically relevant issue in tolerance induction by chimerism. Historically the VCA was performed in established chimeras about 1-2 month after BMT [51, 65, 68] and termed a sequential model (Figure 1). The delay between BMT to solid organ transplantation is clinically applicable in the case of living donor organ transplantation. However, this would not be the case in VCA transplants because the VCA is always a clinical scenario of deceased donor donation setting. The clinically relevant VCA model would be that BMT and VCA are performed simultaneously or the chimerism established after recovery from the VCA. The feasibility of this simultaneous BMT and VCA model is established in rat models. Prabhune et al. reported in an ablative conditioning model that tolerance to hind-limb transplants can be established through the simultaneous transplantation of hind limb and BM in recipients [65]. The rat simultaneous BMT and VCA can also be successfully performed with reduced-intensity nonmyeloablative conditioning (Figure 1. Simultaneous model) (Xu et al., submitted to *Transplantation* 2012) which is closer to the clinical reality. Although simultaneous HSCT-induced mixed chimerism offers an opportunity for tolerance induction, there are obvious drawbacks that would prevent its clinical application. The major concern is that simultaneous BMT and VCA may increase the risk of complications from combined major operation and conditioning for BMT. Moreover, nonmyeloablative conditioning regimens usually require a period of time from days to condition the recipient, which takes five to six days. Attempts to compress the conditioning to ≤2 days would result in unacceptable toxicities. As such, an approach to establish chimerism electively after recovery following VCA (Figure 1. delayed tolerance induction model) using frozen BMC is of critical importance, as the living donor transplant is not clinically feasible for VCA. To address this concern, Chen et al. explored a delayed tolerance induction approach in which BMT was performed 2 months following VCA [82]. They found that donor-specific tolerance can be successfully achieved in VCA when HSCT was performed electively after full recovery from the VCA transplant. The major concern in delayed tolerance induction is that the recipient may become sensitized to donor alloantigens as a result of a prior transplant and may be more prone to BMC rejection [83, 84]. More conditioning and a higher dose of donor BMC are required for engraftment in sensitized recipients as preformed antidonor antibodies contribute as a dominant barrier for the survival of donor cells [85–87]. However, they found that the continuous immunosuppression after VCA and before BMT prevented the generation of antidonor antibodies and effector/memory T cells [82]. This has ensured the success of subsequent donor BMT. The delayed tolerance approach is now being translated to the clinic in an FDA and IRB approved study in living donor kidney transplant recipients.

### 5.5. The Mechanisms of Tolerance Induction by Mixed Chimerism

The pluripotent hematopoietic stem cells engraft and co-exist with recipient stem cells to give rise to all hematopoietic lineages in the recipient. Mixed chimerism is a hybrid immune system. The mutual tolerance in this hybrid immune system must be systemic as suggested by stable and durable coexistence of genetically different donor and recipient hematopoietic components in mixed chimerism. The mutual tolerance in mixed chimeras should be systemic including adaptive immune tolerance (T and B cells) and innate immune tolerance. T-cell tolerance in mixed chimeras has been well studied *in vivo* and *in vitro. *The mechanism of T-cell tolerance is through central deletional mechanisms, in which the allo-activated T cells are deleted by negative selection in the thymus [59, 88]. Functional donor-specific T-cell tolerance has been detected in *in vivo* MLR assays as lymphocytes from mixed chimeras specifically did not respond to host and donor alloantigens, but are competent to respond to genetically disparate third-party alloantigen. Although donor antigens are continuously presented in mixed chimeras, the recipients do not generate antidonor antibody, and vice versa. These data indicate that B-cell tolerance is established in mixed chimeras [89, 90]. T-cell-dependent B-cell immune responses should serve as the mechanism of humoral tolerance [91–93]. As activated T cells are deleted by negative selection in mixed chimeras, there are no donor-specific antigen activated T cells in the periphery to interact with B cells as B cell-activation uniquely requires interaction with activated helper CD4^+^ T cells. The general innate immune tolerance in mixed chimeras is evidenced in an *in vivo* cytotoxicity assay where similar cytotoxicity to donor cells (16.4% ± 8.7%) and to syngeneic cells (9.9% ± 0.8%) occurred and significant cytotoxicity to third-party cells (72.3% ± 3.4%, *P* < 0.005) was detected in mixed chimeras [63]. These results suggest donor-specific innate immune tolerance is achieved in mixed chimeras as the effectors mediating BMC rejection at the early time (<3 days) are innate immune cells.

### 5.6. Preferential Localization and Persistence of Chimerism in Transplanted Donor Bone

Mixed chimerism achieved by nonmyeloablative conditioning has been shown to induce donor-specific tolerance in fully MHC-mismatched VCA recipients. In a paper published by Rahhal et al. [51], WF recipients conditioned with 400 to 100 cGy TBI, transplanted with 100 × 10^6^ T-cell-depleted ACI donor bone-marrow cells, and treated tacrolimus, antilymphocyte serum, and anti-*αβ*TCR showed between 1.8% and 35% donor chimerism 1 month after BMT. Donor engraftment was multilineage in these chimeric recipients 1 to 2 months after BMT. Chimeric animals were then subjected to heterotopic osteomyocutaneous flap transplantation 4–6 weeks after BMT. Over 57% of animals conditioned with 400 cGy TBI and 33% of animals conditioned with 300 cGy TBI showed long-term VCA acceptance though peripheral blood chimerism was lost 5 months after BMT. Interestingly, when donor chimerism was analyzed in various hematopoietic compartments in long-term VCA acceptor animals, there was significantly higher donor chimerism detected in the transplanted donor bone (15.7% ± 4.5%, *P* = 0.0079), recipient bone (4.2% ± 1.0%, *P* = 0.004), spleen (3.1% ± 0.91%, *P* = 0.011), mesenteric lymph node  (1.6 ± 0.47%, *P* = 0.014), and thymus (1.6% ± 0.60%, *P* = 0.036) compared to peripheral blood (0.09% ± 0.06%). The highest level of donor chimerism was detected in the transplanted donor bone. The authors hypothesized that the donor bone may either serve as a tolerizing source of donor lymphoid cells for systemic microchimerism as previously observed [94] or have no underlying effect as tolerance may have been induced at the time of donor BMT. In any event, loss of peripheral blood chimerism did not affect long-term VCA graft acceptance suggesting a role for microchimerism in peripheral blood. Under conditions of low donor chimerism, regulation of immune responses can be maintained by other mechanisms involving regulatory T cells (T_reg_) [95].

## 6. Role of T Regulatory Cells in Long-Term Allograft Acceptance

**Figure 2:**
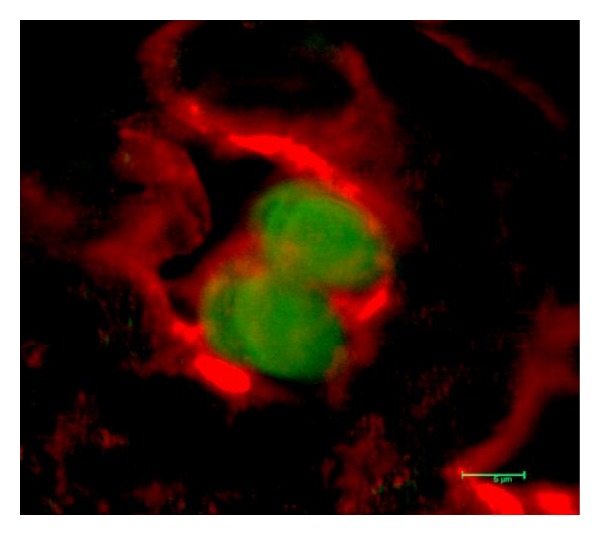
Skin sample from long-term WF-ACI VCA acceptor animals stained for CD4 (red) cell surface staining and FoxP3 (green) intracellular staining. The merged image shows CD4^+^/FoxP3^+^ cells.

CD4^+^ CD25^+^/FoxP3^+^  T_reg_ play a principal role in regulating immune responses to allogeneic antigens and are robust suppressors of T-cell activation [96]. Using a similar rat model, Bozulic et al. [97] evaluated the function of T_reg_ in peripheral tolerance to VCA. WF recipients were conditioned with 400 cGy TBI, transplanted with 100 × 10^6^ T-cell-depleted ACI donor bone-marrow cells, and treated with tacrolimus, anti-lymphocyte serum, and anti-*αβ*TCR. Recipients were monitored for engraftment and then transplanted with a heterotopic osteomyocutaneous flap. Peripheral blood donor chimerism at 1 month after BMT was approximately 30% and was multilineage for both lymphoid and myeloid cells. Sixty-seven percent of transplanted animals displayed long-term acceptance. The group demonstrated that sorted CD8^−^CD4^+^/CD25^+^  T_reg_ from spleens of VCA transplanted animals could significantly suppress cell proliferation when plated in a 1 : 1 ratio with either WF responders/ACI stimulator or WF responder/F344 stimulator. Interestingly, when these sorted cells were restained for FoxP3^+^  T_reg_, VCA rejector animals demonstrated higher absolute numbers of FoxP3^+^  T_reg_. Similarly, Bunnag et al. showed higher levels of FoxP3 mRNA levels in rejected human renal tissue compared to nonrejected tissue [98]. In addition, there was a 12-fold increase in the absolute number of recipient-derived FoxP3^+^  T_reg_ 6 months after-CTA which suggested a potential role for T_reg_ in peripheral tolerance whereby newly induced T_reg_ could potentially migrate to target tissue in high numbers as needed. Because FoxP3^+^ cells were detected in the peripheral blood and appeared to increase over time, immunofluorescent assays were carried out to investigate the presence of FoxP3^+^  T_reg_ at the VCA graft site. Though FoxP3^+^  T_reg_ were not detected in skin samples from VCA-rejected animals, skin samples from long-term VCA acceptor animals stained positive for CD4^+^FoxP3^+^  T_reg_ (Figure 2). Recently, FoxP3^+^  T_reg_ were detected in biopsies from human hand allografts [99] and in human hand transplants undergoing severe rejection [100]. To confirm that the detection of FoxP3^+^ cells in the VCA acceptor animals was not due to the VCA transplant itself, syngeneic controls were performed where WF rats were conditioned in a similar manner but received WF bone-marrow cells and a WF CTA. No CD4^+^FoxP3^+^ cells were detected in the transplanted graft samples from these animals.

The lack of FoxP3^+^  T_reg_ expression in the skin of CTA-rejector animals correlated with the increase in FoxP3^+^  T_reg_ expression in the spleen of rejected animals. Lu et al. showed elevated numbers of mast cells and T_reg_ in tolerant skin grafts in a donor-specific transfusion/anti-CD154 model [101]. Similarly, Mathes et al. demonstrated increased numbers of CD3^+^FoxP3^+^  T_reg_ in the skin and muscle of tolerant composite allografts [102]. However, FoxP3^+^  T_reg_ have also been detected in rejected allografts. Biopsies from human hand transplant recipients undergoing severe rejection demonstrated elevated FoxP3^+^  T_reg_ [100]. In addition, increased numbers of T_reg_ were detected in skin biopsies from patients with acute GVHD compared to patients without GVHD [103]. Time-course studies suggest that T_reg_ are recruited to the site of antigenic challenge early after transplantation to effectively prevent the infiltration of effector T cells [104]. Similarly, Chauhan et al. showed that T_reg_ suppress the induction phase of immune responses in draining lymph nodes rather than the effector phase in the periphery [105]. As such, there may exist a pool of FoxP^+^  T_reg_ that home to various tissue sites as needed to induce tolerance early on after antigenic challenge and then maintain peripheral tolerance. Recently, Hoerning et al. demonstrated that circulating CD4^+^FoxP3^+^CXCR3^+^  T_reg_ correlate with renal allograft function and that peripheral immunoregulation depends on T_reg_ allograft homing [106]. In addition, the timing of acquired biopsies may account for when FoxP3^+^  T_reg_ are detected in tolerant or rejected tissues. Bunnag et al. showed that in human renal transplants, FoxP3 expression increased with time after-transplant. As such, late biopsies had greater FoxP3 expression than early biopsies [98]. Taken together, both time and location of infiltrating FoxP3^+^  T_reg_ may be important in tolerance induction and long-term VCA graft survival. 

## 7. The Future of VCA

Significant progress has occurred in VCA in the past decade. Alexis Carrel, Peter Medawar, and Joseph Murray would be pleased to find that their pioneering work would one day make hand and face allotransplantation a reality. However, the next major advance to make VCA widely available is to minimize or avoid the toxicities of the immunosuppressive agents altogether. Based on the recent clinical success in renal transplantation, tolerance induction may be a path ahead. Future research should focus on establishing safe, simple, and durable donor-specific tolerance in HLA-mismatched recipients of VCA. Drug-free graft approaches to achieve acceptance have been termed the “holy grail” in transplantation and would represent a transformational achievement for VCA to reconstruct traumatic combat-related and civilian injuries, allowing unlimited tissue for repair.
